# SIRT1-dependent mitochondrial biogenesis supports therapeutic effects of resveratrol against neurodevelopment damage by fluoride

**DOI:** 10.7150/thno.42387

**Published:** 2020-03-26

**Authors:** Qian Zhao, Zhiyuan Tian, Guoyu Zhou, Qiang Niu, Jingwen Chen, Pei Li, Lixin Dong, Tao Xia, Shun Zhang, Aiguo Wang

**Affiliations:** 1 Department of Occupational and Environmental Health, School of Public Health, Tongji Medical College, Huazhong University of Science and Technology, Wuhan, Hubei, People's Republic of China; 2 Key Laboratory of Environment and Health, Ministry of Education & Ministry of Environmental Protection, State Key Laboratory of Environmental health (incubating), School of Public Health, Tongji Medical College, Huazhong University of Science and Technology, Wuhan, Hubei, People's Republic of China; 3 Department of Environmental Health, College of Public Health, Zhengzhou University, Zhengzhou, Henan, People's Republic of China

**Keywords:** fluoride, neurodevelopmental damage, mitochondrial biogenesis, SIRT1, resveratrol

## Abstract

**Rationale**: Potential adverse effects of fluoride on neurodevelopment has been extensively explored and mitochondria have been recognized as critical targets. Mitochondrial biogenesis serves a crucial role in maintaining mitochondrial homeostasis and salubrious properties of resveratrol (RSV) has been well-defined. However, the molecular mechanisms governing mitochondrial biogenesis in developmental fluoride neurotoxicity remain unclear and the related therapeutic dietary agent is lacking.

**Methods**: *In vitro* neuroblastoma SH-SY5Y cells and *in vivo* Sprague-Dawley rat model of developmental fluoride exposure were adopted. A total population of 60 children under long-term stable fluoride exposure were also recruited. This work used a combination of biochemical and behavioral techniques. Biochemical methods included analysis of mitochondrial function and mitochondrial biogenesis, as well as mRNA and protein expression of mitochondrial biogenesis signaling molecules, including silent information regulator 1 (SIRT1), peroxisome proliferator-activated receptor γ coactivator-1α (PGC-1α), nuclear respiratory factor 1 (NRF1) and mitochondrial transcription factor A (TFAM). Behavioral studies investigated spatial learning and memory ability of rats.

**Results**: Both *in vivo* and *in vitro* experiments showed that sodium fluoride (NaF) caused mitochondrial dysfunction and impaired mitochondrial biogenesis. Also, NaF elevated SIRT1 levels and suppressed SIRT1 deacetylase activity along with decreased levels of PGC-1α, NRF1 and TFAM, suggestive of dysregulation of mitochondrial biogenesis signaling molecules. Moreover, enhancement of mitochondrial biogenesis by TFAM overexpression alleviated NaF-induced neuronal death through improving mitochondrial function *in vitro*. Further *in vivo* and *in vitro* studies identified RSV, the strongest specific SIRT1 activator, improved mitochondrial biogenesis and subsequent mitochondrial function to protect against developmental fluoride neurotoxicity via activating SIRT1-dependent PGC-1α/NRF1/TFAM signaling pathway. Noteworthy, epidemiological data indicated intimate correlations between disturbed circulating levels of mitochondrial biogenesis signaling molecules and fluoride-caused intellectual loss in children.

**Conclusions**: Our data suggest the pivotal role of impaired mitochondrial biogenesis in developmental fluoride neurotoxicity and the underlying SIRT1 signaling dysfunction in such neurotoxic process, which emphasizes RSV as a potential therapeutic dietary agent for relieving developmental fluoride neurotoxicity.

## Introduction

Optimal fluoride concentration range (<=1.0 mg/L) in drinking water is recommended to be safe to human body but excess fluoride content (above this limit) is considered deleterious to health 1. Indeed, drinking-water-type fluorosis is main type of endemic fluorosis which has been documented in China, Iran, India and so on 2-4. Of particular note is that potential adverse effects of fluoride on neurodevelopment have attracted considerable attention. Multiple epidemiological studies of developmental fluoride neurotoxicity exhibited an inverse correlation between high fluoride exposure and children's intelligence 5. Laboratory studies further demonstrated developmental exposure to fluoride adversely affects neuron function including learning and memory impairment 6, 7. However, additional research is still warranted to reveal mechanisms underlying developmental fluoride neurotoxicity.

To date mitochondria have been well-accepted as critical targets, which is clearly demonstrated by a stereological study of fetal brain samples that exposure to fluoride caused mitochondrial impairment 8. Additionally, mitochondrial damages, like cristae disorder, membrane potential loss and imbalanced fusion/fission status, were reported *in vivo* and *in vitro* neuronal cell lines exposed to fluoride 9, 10. Although numerous studies have established an association between developmental fluoride neurotoxicity and mitochondrial abnormalities, the detailed mechanisms underlying this process still remain largely unclear.

During the life cycle of mitochondria, mitochondrial biogenesis assumes a predominant role in maintaining mitochondrial homeostasis to meet cellular physiological needs 11. Mitochondrial biogenesis is a tightly regulated process that involves the coordinated action of both nuclear and mitochondrial genomes 12. Peroxisome proliferator-activated receptor γ coactivator-1α (PGC-1α), a major modulator, can interact with nuclear respiratory factor 1 (NRF1), together inducing transactivation of many genes whose products are imported into mitochondria, including mitochondrial transcription factor A (TFAM), to encode mitochondria-specific proteins 11. Strong research evidence offers that impaired mitochondrial biogenesis potentially contributes to the disruption of mitochondrial function and takes a critical role in the pathogenesis of neurodegenerative diseases 13. Nonetheless, whether perturbed mitochondrial biogenesis contributes to impaired neurodevelopment induced by fluoride has not been clearly elaborated.

Resveratrol (trans-3, 4', 5-trihydroxy stilbene, RSV), is a plant-derived polyphenolic compound belonging to a class of stilbenes, which has been widely found in many plant species including grapes, berries, peanuts and herbs 14. It is known that RSV is a potent natural activator of silent information regulator 1 (SIRT1), which is an NAD^+^-dependent protein deacetylase 15. SIRT1 activation is able to enhance PGC-1α activity, promoting mitochondrial biogenesis and thus maintaining mitochondrial function 16. Noteworthy, RSV has been documented to be neuroprotective against several neurotoxic compounds 17, 18. And a wide range of scientific evidence showed the major neuroprotective mechanisms of RSV were associated with SIRT1 activation 19. Although RSV's ability to suppress toxicity of fluoride has also been widely publicized 20-22, however, the evidence in support of RSV protection in developmental fluoride neurotoxicity, especially the role of SIRT1 in this process, urgently needs further explorations.

Therefore, in the present study, we aimed to evaluate the role of mitochondrial biogenesis in developmental fluoride neurotoxicity using *in vitro* SH-SY5Y cell culture and *in vivo* Sprague-Dawley rat model of developmental fluoride exposure from pre-pregnancy until 2 months of delivery. Particularly, we investigated the protective role of resveratrol in developmental fluoride neurotoxicity *in vivo* and *in vitro*. Furthermore, the physiological relevance of mitochondrial biogenesis in children under stable long-term exposure to drinking-water fluoride was also investigated.

## Methods

### Cell culture and treatments

Human neuroblastoma SH-SY5Y cells were obtained from American Type Culture Collection (ATCC Inc., Manassas, VA, USA) and routinely cultured in Dulbecco's modified eagle's medium/ Ham's F-12 (Gibco, Thermo Fisher Scientific Inc., Waltham, Massachusetts, USA), supplemented with fetal bovine serum (10%, Gibco) at 37 °C under humidified 5% CO_2_ atmosphere. Appropriate cell density was used in accordance with the different assays we performed.

When at 35-60 passages, cells in exponential growth phase were exposed to different concentration of sodium fluoride (NaF, Sigma-Aldrich CO., St Louis, MO, USA) for 24 h. NaF was freshly prepared as 4 g/L stock solutions in distilled water and the dosages in the present experiment were selected as 20, 40 and 60 mg/L (namely 0.476, 0.952 and 1.429 mM, respectively) based on the cell viability assay in our previous study 23. The recombinant adenovirus with TFAM overexpression (Ad-TFAM, MOI = 100) and control adenovirus (Vector) were constructed by Vigene Biosciences (Shandong, China). Cells were exposed to 60 mg/L NaF after infected with ad-TFAM for 24 h. In addition, cells were exposed to 20 μM resveratrol (RSV, Sigma-Aldrich) for 2 h prior to NaF treatment or NaF and 3 mM nicotinamide (NIC, Sigma-Aldrich) co-stimulation for 24 h. The concentration and time period of interference agents were based on concentrations in previous studies in SH-SY5Y cells 24, 25, as well as our preliminary trials. No FBS was present in culture medium when cells were treated with NaF or/and interference agents mentioned above.

### Animals and experimental designs

Adult Sprague-Dawley rats, weighing 180-200 g, were purchased from Laboratory Animal Center of Hubei Provincial Center for Disease Control and Prevention. The protocol for the study was approved by the Ethics Review Committee for Animal Research at Huazhong University of Science and Technology and all experiments were performed in accordance with the Guide for the Care and Use of Laboratory Animals published by Ministry of Health People's Republic of China.

Fluoride ion concentration in drinking water in fluorosis areas is higher than 0.5 mg/L and up to 48 mg/L 26. Considering environmental fluoride levels in drinking water and the fact rodents are more efficient at excreting fluoride from their bodies than humans, rats were divided into 4 groups: Control group (tap water, containing less than 1.0 mg/L fluoride ion), different concentration of NaF group (10, 50 and 100 mg/L, namely 0.238, 1.19 and 2.381 mM, corresponding to 4.52, 22.6 and 45.2 mg/L fluoride ion, respectively) to practically mimic the real children exposure in the natural environment as far as possible.

**Figure 1:**
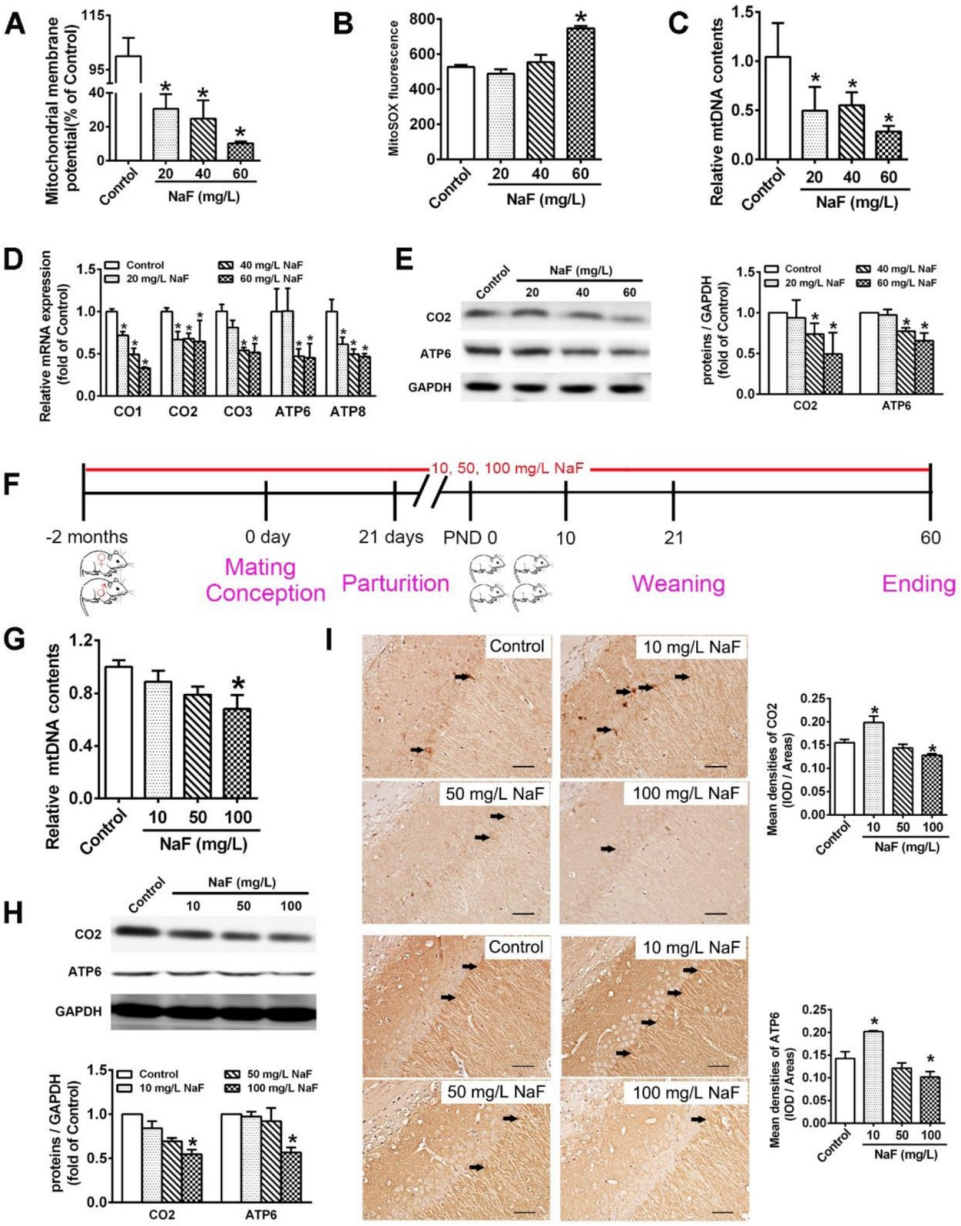
** NaF causes mitochondrial dysfunction and mitochondrial biogenesis impairment *in vitro* and *in vivo*. (A, B)** MMP levels (A) and mitoROS production (B) in SH-SY5Y cells determined by flow cytometry. **(C, D)** RT-qPCR analyses of relative mtDNA contents (C) and representative mtDNA-encoded genes including CO1, CO2, CO3, ATP6, ATP8 (D) in SH-SY5Y cells. Quantification represents the levels of the indicated ND4 and mRNA normalized to GAPDH. **(E)** Immunoblot analyses of CO2 and ATP6 in SH-SY5Y cells. Quantification represents the levels of the indicated protein normalized to GAPDH. **(F)** Experimental designs of a cohort of offspring rats subjected to NaF treatments with different concentrations. **(G)** RT-qPCR analyses of relative mtDNA contents in hippocampal tissues (*n* = 6 rats per group). **(H)** Immunoblot analyses of CO2 and ATP6 in hippocampal tissues (*n* = 3 rats per group). **(I)** Representative images of the IHC staining for CO2-expressing (CO2^+^) and ATP6-expressing (ATP6^+^) neurons in hippocampal CA1 region. CO2^+^ and ATP6^+^ neuronal cells are demonstrated by black arrows and quantified. Scale bars represent 50 μm, *n* = 2 rats per group. SH-SY5Y cells were treated with different concentrations of NaF (20, 40 and 60 mg/L). Offspring SD rats were developmentally exposed to NaF (10, 50 and 100 mg/L) from pre-pregnancy until 2 months of delivery. Data information: Data are presented as mean ± SD. Data were cumulative of at least three independent experiments (A-E). * *P* < 0.05 is considered significant compared with Control by one-way ANOVA test.

In study-1, female rats were developmentally exposed to fluoride via drinking water freely daily from pre-pregnancy to post-puberty, which covers the critical maternal, perinatal, and pubertal periods. The day of parturition was counted as postnatal day 0 (PND 0). Offspring female rats were still under the same treatments as mother rats till PND60 after the weaning period (PND21) (Figure 1F).

In study-2, offspring female rats were allotted into 4 different groups (*n* = 20 pups in each group). The treatment schedule was given below (Figure 6A):

- Group I — Control (receiving tap water only till PND 60).
- Group II — Fluoride-treated (100 mg/L NaF via drinking water till PND 60).
- Group III — RSV supplemented (100 mg/L NaF via drinking water till PND 60 + RSV at 200 mg/kg body weight/day by gavage from PND 10 to PND 60).
- Group IV — RSV and NIC supplemented (NaF at a dose of 100 mg/L via drinking water till PND 60 + RSV at 200 mg/kg body weight/day and NIC at 100 mg/kg body weight/day simultaneously by gavage from PND 10 to PND 60).

After corresponding treatments, all offspring rats were euthanized by cervical dislocation for the study of biochemical parameters.

### Study population

Baodi district in Tianjin, China is divided into historical high fluoride areas and normal fluoride areas. In 2015, the volunteers aged 8-12 years were selected from local children who are permanent residents since birth. Children from Dakoutun town (high-fluoride districts, *n* = 30) and students from Lintingkou town (normal-fluoride/control areas, *n* = 30) were included in this study. Both the two study sites were not in the endemic areas for iodine deficiency disorders, or exposed to other potential neurotoxins like lead, arsenic or mercury. This study was approved by the Review Board of Huazhong University of Science and Technology and Ethical Committee of Tianjin Center for Disease Control and Prevention. As all the research participants were minors, written informed consent was obtained from all the participants and their parents/guardians before study enrollment.

### Western blot analysis

BCA protein assay kits (Beyotime Institute of Biotechnology, Nantong, Jiangsu, China) were used to assess concentrations of protein extracted from hippocampi and cell cytosol. A total of 30 μg of proteins denatured was loaded on to gels, separated using SDS-PAGE and transferred onto polyvinylidene fluoride (PVDF) membranes (Roche Inc., Nutley, New Jersey, USA). Following blocked with 5% skim milk, the membranes were incubated overnight at 4 °C with primary antibodies against SIRT1 (1:1000, Proteintech Group, Inc., Chicago, IL, USA), PGC-1α (1:1000, Abcam Inc., Cambridge, MA, USA), NRF1 (1:10000, Abcam Inc.), TFAM (1:1000, Proteintech Group, Inc.), CO2 (1:1000, Proteintech Group, Inc.), ATP6 (1:2000, Proteintech Group, Inc.), and GAPDH (1:8000, Bioworld Technology Inc., St Louis Park, MN, USA). Subsequently, the membranes were incubated with HRP-conjugated goat anti-mouse IgG or goat anti-rabbit IgG for 1 h at room temperature. Finally, the signals of membranes were determined using ECL reagents (Advansta Inc., Menlo Park, CA, USA) and scanned with the GeneGnome chemiluminescent imaging system (Syngene Inc., Frederick, MD, USA). The band intensities were quantified by Quantity One (Bio-Rad, Hercules, CA, USA).

### Reverse transcription-quantitative real-time PCR (RT-qPCR)

Total RNA was isolated with TRIzol reagent (Invitrogen Corp.) and then converted into cDNA using a Revert Aid First Strand cDNA Synthesis Kit (Fermentas Inc., Hanover, MD, USA). cDNA products were stored at -80 °C until used. The RT-qPCR was performed using an ABI PRISM 7900 HT PCR system (Applied Biosystems; Thermo Fisher Scientific, Inc., Waltham, MA, USA) and SYBR Green PCR Master Mix reagent kits (Roche, Inc., Nutley, New Jersey, USA). Detailed reaction conditions were as follows: 95 °C for 10 min, 40 cycles of 95 °C for 15 s and 60 °C for 1 min. Primer sequences were listed in Table S1 and synthesized by Sangon Biotech Co. (Shanghai, China). SDS 2.2.1 system software was employed to analyze the fluorescence threshold values and quantitative calculations were performed using 2**^-^**^ΔΔCt^ method 27.

### Chromatin immunoprecipitation-polymerase chain reaction (ChIP-PCR)

ChIP assay was carried out using the ChIP assay kit (Beyotime Institute of Biotechnology) as described previously 28. In brief, SH-SY5Y cells were crosslinked with 1% formaldehyde and then quenched with glycine. After the process of sonication at 10 s/time for 15 times, sheared chromatins (input) were immunoprecipitated with anti-PGC-1α, anti-NRF1 and anti-TFAM antibodies respectively, followed by incubation with Protein A+G Agarose/Salmon Sperm DNA. 2 μl of each of the purified chromatin-immunoprecipitated DNA was used as template for 35 cycles of PCR amplification. The PCR products were analyzed on 1.5% agarose gel. The primers possible specific to the PGC-1α, NRF1 and TFAM binding regions within SIRT1 promoter were as follows: 5'-AACGGCTAGATAGCTCACGC-3'; 3'-CCGATCTACTTTCTGGCCCC-5'. IgG (Cell Signaling Technology, Inc., Danvers, MA, USA) was served as the negative control.

### Flow cytometry

To examine mitochondrial superoxide (mitoROS), treated cells were harvested and stained with MitoSOX™ Red agent (5 μM, Invitrogen Corp.) for 10 min at 37 °C. Then cells were washed twice with PBS, resuspended in PBS, and examined by FACSCalibur flow cytometry system (BD Biosciences, San Jose, CA, USA). Ex/Em was set as 510/580 nm. The relative mean fluorescence intensities were analyzed using FlowJo software version 10 (TreeStar Inc., Ashland, OR, USA).

Mitochondrial membrane potential (MMP) in treated cells were measured by JC-10 (20 μM, Invitrogen Corp.). After JC-10 incubation for 30 min at 37 °C, monomeric green fluorescence emissions and aggregate red fluorescence intensities in cells were monitored at Ex/Em = 490/525 nm by flow cytometry. The MMP in each group was calculated as the fluorescence ratio of red to green and expressed as a relative ratio of the level in the control group.

### Mitochondrial DNA (mtDNA) content

The total DNA from hippocampal specimens and cell lysis were isolated using DNA extraction kits (Tiangen Biotech Co., Beijing, China). Primer sequences were listed in Table S2 and synthesized by Sangon Biotech Co. Mitochondrial and nuclear DNA (nDNA)were assessed by RT-qPCR respectively and mtDNA to nuclear DNA ratio were adopted as relative mitochondrial contents. ND4 and GAPDH was chosen to represent mtDNA and nDNA.

### Cell viability assay

Cell viability was analyzed using a cell counting kit-8 (CCK-8, Proteintech Group, Inc.). In 96-well culture plates, a mixture of 10 μl of medium and 10 μl of CCK-8 solution were added to each well at the end of exposure and the cells were incubated for 2 h at 37 °C. The absorbances at 450 nm were measured using Synergy 4 multifunctional microplate reader (BioTek Instruments Inc., Winooski, VT, USA). The results were expressed as a percentage of the values of the untreated control set as 100%.

### SIRT1 deacetylase activity detection

SIRT1 deacetylase activity was quantified with a SIRT1 assay kit (Sigma-Aldrich). Briefly, proteins were extracted and the fluorescence intensities were measured at Ex/Em = 340/440 nm with a Synergy 4 multifunctional microplate reader (BioTek Instruments Inc.). Experimental values were represented as a percentage of control.

### Morris water maze (MWM) test

In the MWM test, water was made opaque by the addition of black non-toxic ink. During the place navigation test, each rat was allowed a maximum of 60 s to find the hidden platform for the first 4 consecutive days, 4 trials per day with a 30 s interval. On each trial, the rats started from one of the middle of the four quadrants. Escape latencies and swimming patterns (swimming speed and path length to find the hidden platform) were digitally monitored. On the 5th day, the spatial exploration test was performed, in which rats were subjected to a 60 s free swim to find the previous location of the platform starting from each of the four quadrants. The number of times that the rats pass through the platform site and the proportion of time and swimming distance that the rats spent searching for the platform were automatically recorded. The pathway that the rats passed through the previous platform quadrant was recorded by a video camera which was connected to a digital tracking device attached to the computer loaded with the water maze software (Electric factory of Wuhan, Hubei, China).

### Transmission electron microscopy (TEM) examination

The hippocampal samples were cut into small pieces (0.8-1.0 mm^3^) and fixed with 2.5% pre-cooling glutaraldehyde for 6 h at 4 °C. After that, these samples were dehydrated by graded ethanol and acetone, infiltrated with a mixture of one-half propyleneoxide and embedded in resin. Then a 50 nm-thick section were cut and collected onto grids. Finally, grids were stained with 4% uranyl acetate for 15-30 min and 0.5% ad citrate for 3-15 min. The ultrastructure in hippocampus was observed using transmission electron microscope (Philips Tecnai 10, Philips Co., Eindhoven, the Netherlands).

### Nissl staining

The brains were demineralized by EDTA and embedded in paraffin followed by 4% cold paraformaldehyde overnight for fixing. Specifically, 20 μm-thick sections were successively permeated, rinsed and differentiated based on standard procedures and well-prepared for microscopic examinations. The neurons and Nissl bodies in hippocampal CA1 regions were photographed using Olympus BX53 microscope (Olympus Co., Tokyo, Japan). Image-pro Plus 6.0 (Media Cybernetic Inc., Silver Springs, MD, USA) was used to calculate neuronal cellular number.

### Immunohistochemical (IHC) staining

The hippocampal sections were deparaffinized, rehydrated and immersed successively. Next sections were incubated in 10% goat serum sealing fluid for blocking nonspecific binding sites. Primary antibodies, including anti-SIRT1 (1:200), anti-PGC-1α (1:150), anti-NRF1 (1:50), anti-TFAM (1:200), anti-CO2 (1:50) and anti-ATP6 (1:50), were added overnight at 4 °C. Then sections were incubated with biotin-labeled goat anti-rabbit/mouse IgG working solutions (1:500) and kept for 2 h at room temperature. Subsequently, slides were incubated with StreptAvidin Biotin HRP Complex, colored at room temperature using pre-prepared DAB chromogenic solutions. Thereafter, cells were visualized under a microscope (Olympus Co.) and the protein expression in cells were quantified and analyzed using Image-pro Plus 6.0 software.

### Sample collection and detections

IQ scores were measured using the second edition of Combined Raven's Test-The Rural in China (CRT-RC2) 29. Children completed the test independently under the supervision of our four trained professionals. A total of 5 ml blood extracted from each child was treated with anti-coagulant. Spot (early-morning) urine samples from every child, and drinking water samples from each family were collected in precleaned, labelled polythene tubes. All collected samples were transported to laboratory within 2 h in ice boxes under a freezing condition (temperature < 4 °C). Fluoride concentration in urine samples and drinking water samples were analyzed using the national standardized ion selective electrode method in China 30. Lymphocytes in blood were immediately extracted according to the instruction of lymphocyte separation medium (MP Biomedicals, LLC, Santa Ana, CA) and then stored at -80 °C for further examinations. Circulating levels of mitochondrial biogenesis signaling molecules in lymphocytes were measured using enzyme-linked immunosorbent assays (ELISA) kits according to the manufacturer's protocols (Ji Ning Inc., Shanghai, China).

### Statistical analysis

Results are expressed as mean ± SD for the values from at least three independent experiments. Statistical analyses were performed using one-way analysis of variance (ANOVA) using SPSS 20 software (SPSS Inc., Chicago, IL, USA) and presented by Graph Pad Prism 5 software (GraphPad Software Inc., San Diego, CA, USA). If ANOVA indicated significant differences, Dunnett's test was performed to compare mean values between the two study groups. The levels of fluoride concentration in drinking water and circulating mitochondrial biogenesis signaling molecules in peripheral blood of children were compared by independent samples student's *t*-test. We further utilized Pearson correlation analysis to examine correlations between mitochondrial biogenesis signaling molecules and fluoride concentration as well as IQ scores. A value of *P* < 0.05 was considered as statistically significant.

## Results

### Fluoride induces mitochondrial dysfunction and impairs mitochondrial biogenesis

In our study, we found that treatment of SH-SY5Y cells with different concentration of NaF evidently decreased MMP levels (Figure 1A) and increased mitoROS production (Figure 1B), which demonstrates NaF causes mitochondrial dysfunction in SH-SY5Y cells.

Pathophysiological conditions of mitochondrial biogenesis are thought to potentially conduce to mitochondrial dysfunction 31. Increased mitochondrial biogenesis is directly compatible with augmented mtDNA contents as well as enhanced mitochondrial gene expression, which can be reflected by mitochondrial RNA transcripts and protein synthesis 32. NaF treatment reduced relative mtDNA contents both in SH-SY5Y cells (Figure 1C) and in hippocampi extracted from offspring rats (Figure 1G). Moreover, 3 common subunits in the core structure of complex IV (CO1, CO2, and CO3) and 2 subunits in Complex V (ATP6 and ATP8) are synthesized in the mitochondria 33. NaF treatment reduced the mRNA levels of these subunits encoded by mtDNA in SH-SY5Y cells (Figure 1D). In addition, the protein expression of CO2 and ATP6 were evidently downregulated in NaF-treated cells (Figure 1E) and in hippocampi (Figure 1H). Further, the phenotypic expression of CO2 and ATP6 were also verified by IHC staining in hippocampal CA1 region (Figure 1I). Together, these results indicate that exposure of SH-SY5Y cells and offspring rats to NaF spoiled mitochondrial biogenesis *in vivo* and *in vitro*.

### NaF impedes PGC-1α/NRF1/TFAM signaling pathway

**Figure 2:**
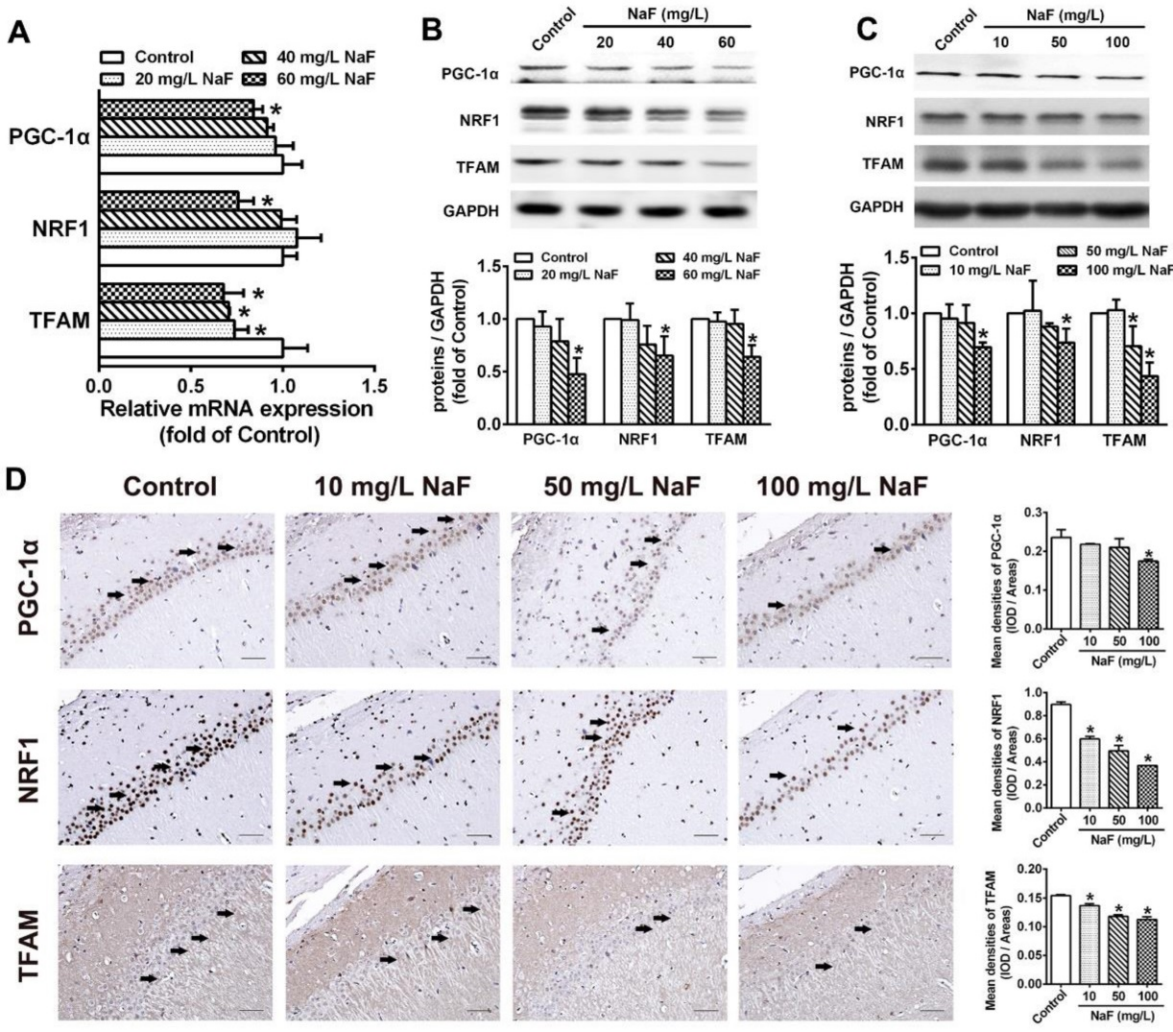
** NaF triggers disruption of mitochondrial biogenesis signaling molecules *in vitro* and *in vivo*. (A, B)** RT-qPCR (A) and immunoblot (B) analyses of PGC-1α, NRF1 and TFAM in SH-SY5Y cells. Quantification represents the levels of the indicated mRNA and protein normalized to GAPDH. **(C)** Immunoblot analyses of PGC-1α, NRF1 and TFAM in hippocampal tissues (*n* = 3 rats per group). **(D)** Representative images of the IHC staining for PGC-1α-expressing (PGC-1α^+^), NRF1-expressing (NRF1^+^) and TFAM-expressing (TFAM^+^) neurons in hippocampal CA1 region. PGC-1α^+^, NRF1^+^ and TFAM^+^ neuronal cells are demonstrated by black arrows and quantified. Scale bars represent 50 µm, *n* = 2 rats per group. Data information: Data are presented as mean ± SD. Data were cumulative of at least three independent experiments (A-B). * *P* < 0.05 is considered significant compared with Control by one-way ANOVA test.

PGC-1α/NRF1/TFAM signaling pathway plays a key role in regulating mitochondrial biogenesis 34, therefore we estimated whether above mentioned mitochondrial biogenesis regulating molecules are altered during NaF exposure. In SH-SY5Y cells treated with NaF, total mRNA and protein levels of PGC-1a, NRF1 and TFAM were all evidently reduced (Figure 2A-B). Consistently, the expression of PGC-1a, NRF1 and TFAM protein were decreased in NaF-exposed hippocampal neurons (Figure 2C). Supportively, IHC staining also exhibited NaF-caused decreased phenotypic expression of PGC-1a, NRF1 and TFAM in hippocampal CA1 region (Figure 2D). These data suggest PGC-1α/NRF1/TFAM signaling pathway are impeded by NaF *in vivo* and *in vitro*.

### Enhanced mitochondrial biogenesis by TFAM overexpression reverses NaF-induced neuronal death via promoting mitochondrial function

**Figure 3:**
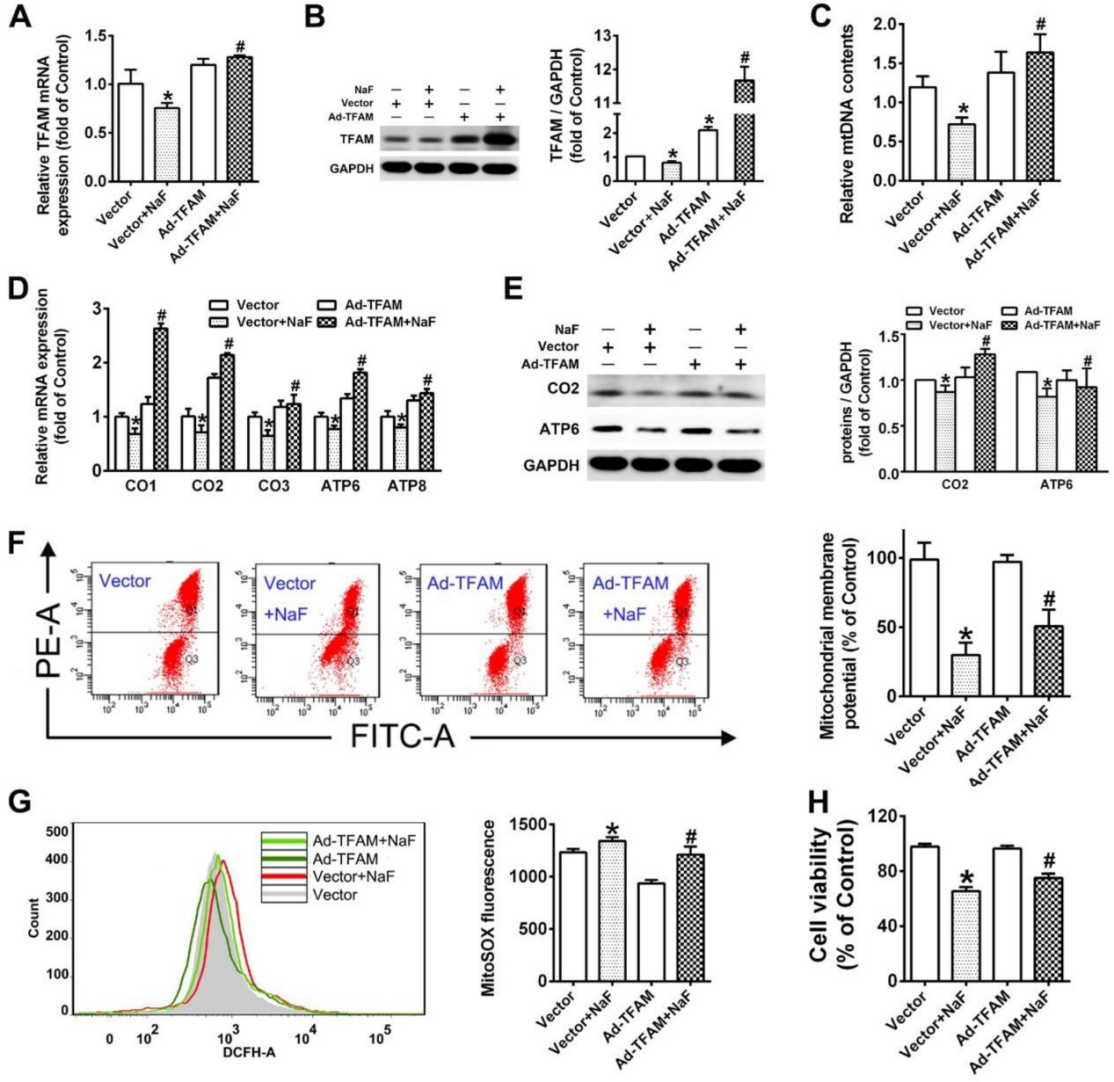
** Improved mitochondrial biogenesis by TFAM overexpression alleviates NaF-induced neuronal death by enhancing mitochondrial function. (A, B)** RT-qPCR (A) and immunoblot (B) analyses of TFAM in SH-SY5Y cells. GAPDH was used as the internal control. **(C)** RT-qPCR analyses of relative mtDNA contents in SH-SY5Y cells. Quantification represents the levels of ND4 normalized to nuclear gene GAPDH. **(D, E)** RT-qPCR (D) and immunoblot (E) analyses of CO1, CO2, CO3, ATP6, ATP8 in SH-SY5Y cells. **(F, G)** Representative flow plots of MMP levels (F) and mitoROS production (G) in SH-SY5Y cells measured by flow cytometry. **(H)** Levels of cell viability in SH-SY5Y cells using CCK-8 assay. SH-SY5Y cells were infected with adenovirus overexpressing TFAM and 24 h later were treated with 60 mg/L NaF for 24 h. Data information: Data are presented as mean ± SD. Data were cumulative of at least three independent experiments. * *P* <0.05 is considered significant compared with Vector group and #*P* < 0.05 is considered significant from Vector+NaF group by one-way ANOVA test.

The well-known TFAM triggers the transcription and replication of mtDNA in the process of mitochondrial biogenesis 35. To assess whether mitochondrial biogenesis is responsible for neurotoxic effects induced by NaF, ad-TFAM were constructed and transfected in SH-SY5Y cells. The stronger mRNA expression and denser immunoreactive band suggested the declined TFAM levels in SH-SY5Y cells exposed to NaF were overexpressed by ad-TFAM transfection successfully (Figure 3A-B). Accordingly, TFAM overexpression reversed the suppressed mitochondrial biogenesis induced by NaF, as manifested by increases in mtDNA contents as well as elevated expression of subunits encoded by mtDNA at both transcriptional and translational levels (Figure 3C-E). Furthermore, TFAM overexpression rescued mitochondrial dysfunction induced by NaF as reflected by increased MMP levels (Figure 3F) along with decreased mitoROS production (Figure 3G). More importantly, TFAM overexpression significantly ameliorated the decline in cell survival caused by NaF (Figure 3H). Thus, these results support enhanced mitochondrial biogenesis by TFAM overexpression alleviate neuronal death induced by NaF through improving mitochondrial function.

### Activity of SIRT1 expression are altered following NaF exposure

**Figure 4:**
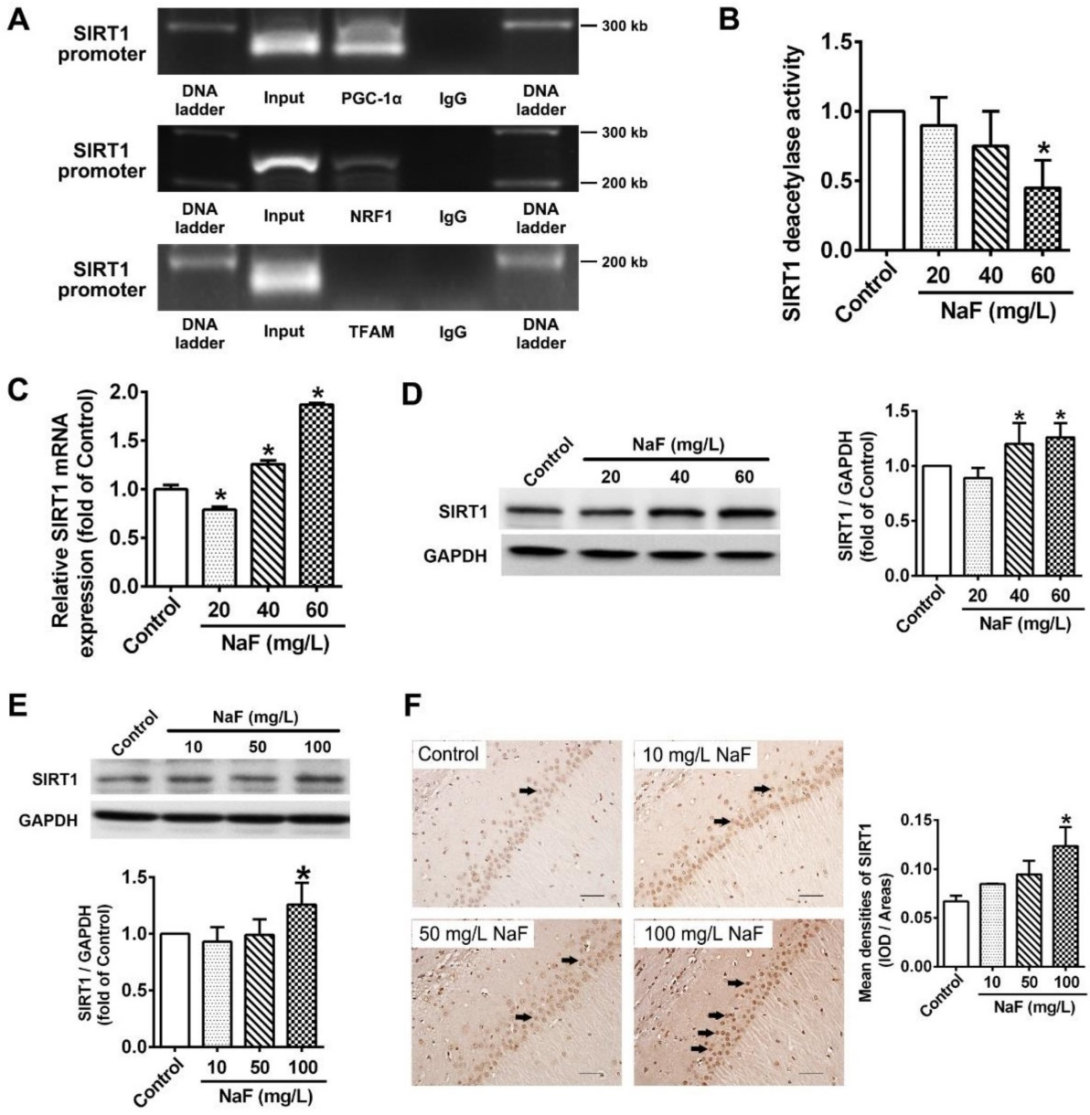
** NaF exposure caused SIRT1 expression changes both *in vitro* and *in vivo*. (A)** ChIP-PCR analyses for PGC-1α, NRF1 and TFAM binding to the SIRT1 promoter in SH-SY5Y cells. **(B)** SIRT1 deacetylase activity in SH-SY5Y cells measured by SIRT1 assay kits. **(C, D)** RT-qPCR (C) and immunoblot (D) analyses of SIRT1 in SH-SY5Y cells. Quantification represents the levels of the indicated mRNA and protein normalized to GAPDH. **(E)** Immunoblot analyses of SIRT1 in hippocampal tissues (*n* = 3 rats per group). **(F)** Representative images of the IHC staining for SIRT1-expressing (SIRT1^+^) neurons in hippocampal CA1 region. SIRT1^+^ neuronal cells are demonstrated by black arrows and quantified. Scale bars represent 50 μm, *n* = 2 rats per group. Data information: Data are presented as mean ± SD. Data were cumulative of at least three independent experiments (A-D). * *P* < 0.05 is considered significant compared with Control by one-way ANOVA test.

Substantial evidence has indicated that SIRT1 interacts with PGC-1a to promote mitochondrial biogenesis and maintain mitochondrial function 36. Therefore, ChIP-PCR was performed in SH-SY5Y cells to elucidate whether SIRT1 transcriptionally regulates PGC-1a, NRF1 and TFAM, which revealed that SIRT1 targeted both PGC-1a and NRF1 except TFAM (Figure 4A). Subsequently, we found that SIRT1 deacetylase activity were significantly declined by NaF (Figure 4B). However, SIRT1 expression at transcriptional and translational level had appreciable increases in NaF-treated cells (Figure 4C-D). Consistently, NaF resulted in an obvious increase of SIRT1 protein levels in NaF-exposed hippocampus (Figure 4E), which were verified by IHC staining (Figure 4F). Taken together, these data suggest NaF exposure causes changes of SIRT1 expression *in vivo* and *in vitro*.

### RSV counteracts NaF-induced mitochondrial dysfunction and neuronal death via activating mitochondrial biogenesis by triggering SIRT1-depentent PGC-1α/NRF1/TFAM signaling pathway in SH-SY5Y cells

**Figure 5:**
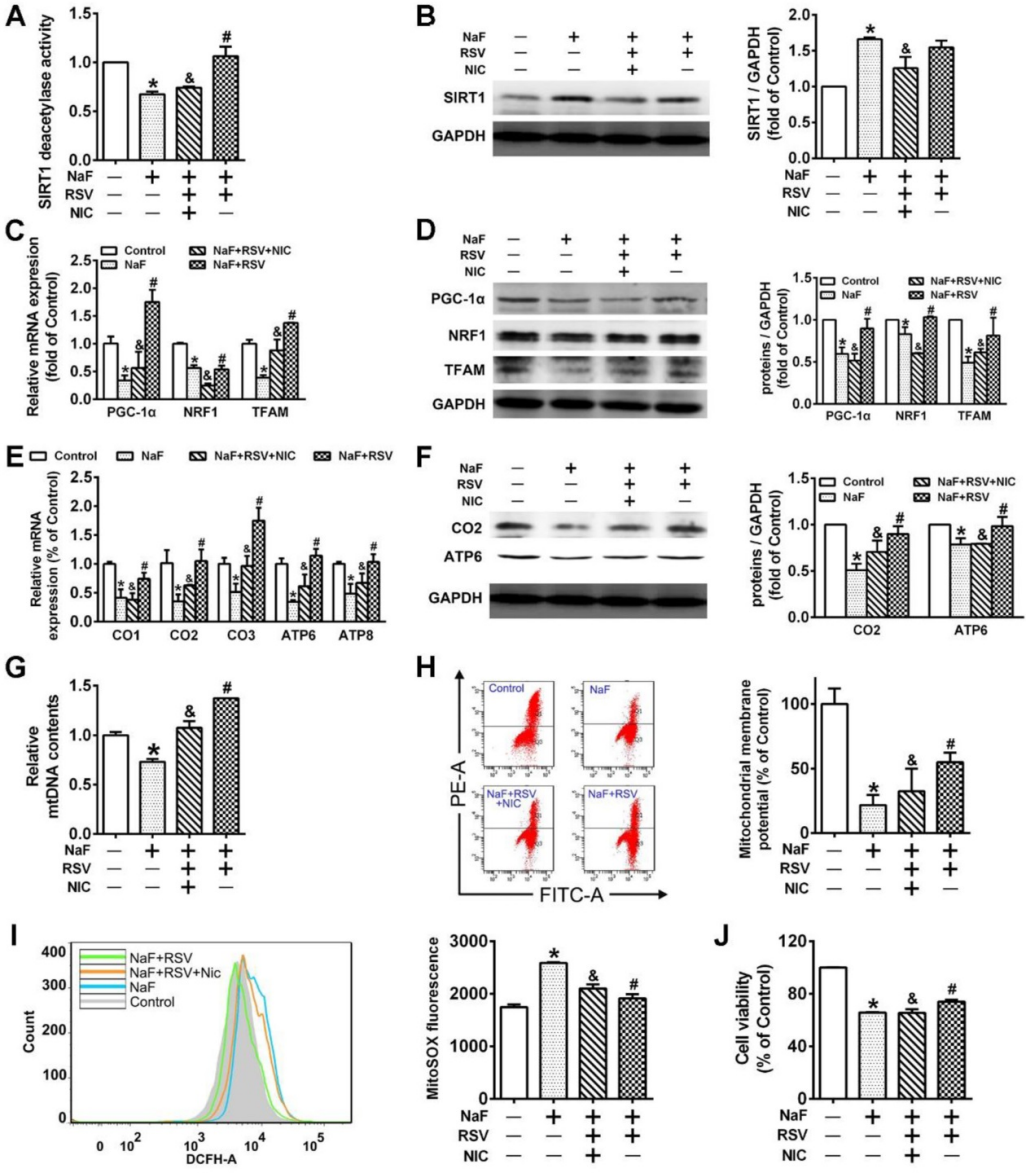
** RSV protects cells from NaF-caused adverse neuronal effects via promoting SIRT1-depeendent PGC-1α-NRF1-TFAM signaling pathway. (A)** SIRT1 deacetylase activity in SH-SY5Y cells using SIRT1 assay kit. **(B)** Immunoblot analysis of SIRT1 in SH-SY5Y cells and the corresponding quantification. **(C, D)** RT-qPCR (C) and immunoblot (D) analyses of PGC-1α, NRF1 and TFAM in SH-SY5Y cells. Quantification represents the levels of the indicated mRNA and protein normalized to GAPDH. **(E, F)** RT-qPCR (E) and immunoblot (F) analyses of representative subunits encoded by mtDNA in SH-SY5Y cells. Quantification represents the levels of the indicated mRNA and protein normalized to GAPDH. **(G)** RT-qPCR analysis of relative mtDNA contents in SH-SY5Y cells. **(H, I)** Representative flow plots of MMP levels (H) and mitoROS production (I) in SH-SY5Y cells using flow cytometry. **(J)** Levels of cell viability in SH-SY5Y cells determined by CCK-8 assay. SH-SY5Y cells were preincubated with 20 μM RSV for 2 h followed by co-culturing with 60 mg/L NaF and 3 mM NIC for 24 h. Data information: Data are presented as mean ± SD. Data were cumulative of at least three independent experiments. * *P* < 0.05 is considered significant compared with Control, ^#^*P* < 0.05 is significantly different from NaF group and ^&^*P* < 0.05 is considered significant from NaF+RSV group by one-way ANOVA test.

RSV's therapeutic properties in neurodegeneration has been widely publicized 37. In this study, we observed an ameliorated cell viability (Figure S1A) and recovery of mitochondrial function (Figure S1B-C) under NaF exposure pretreated with RSV. Through exploring the protective mechanisms of RSV against NaF-induced neurotoxicity, RSV pretreatment reversed the defective mitochondrial biogenesis (Figure S1D-F) and the suppressed expression of related signaling molecules (Figure S1G-H) caused by NaF. As for SIRT1 expression, however, the presence of RSV upregulated the reduced SIRT1 deacetylase activity after NaF treatment (Figure S1I), with the protein expression of SIRT1 unchanged (Figure S1J). Therefore, we then evaluated the effects of SIRT1 on specific pathways in the RSV's neuroprotection using SIRT1 antagonist, NIC. Importantly, we observed not only inhibited SIRT1 deacetylase activity but evidently decreased SIRT1 protein levels in NaF-treated cells with RSV and NIC co-incubation (Figure 5A-B).Furthermore, all the beneficial effects of RSV on fluoride insults were significantly blocked by NIC. Concretely speaking, NIC successfully suppressed the ameliorative effects of RSV on expression of PGC-1α, NRF1 and TFAM at both the transcriptional and translational levels after NaF treatment (Figure 5C-D). Moreover, NIC treatment conferred the opposite shift in RSV's protection for mitochondrial activity towards a more serious impairment in mitochondrial biogenesis (Figure 5E-G) and following deteriorative mitochondrial dysfunction (Figure 5H-I), thus causing a more serious decline in cell viability (Figure 5J). All data suggest RSV protect SH-SY5Y cells from NaF-induced mitochondrial dysfunction and neuronal death through activating mitochondrial biogenesis regulated by SIRT1-dependent PGC-1α/NRF1/TFAM signaling pathway.

### RSV prevents cognitive impairments of offspring rats exposed to NaF through eliciting SIRT1-dependent mitochondrial biogenesis process

**Figure 6:**
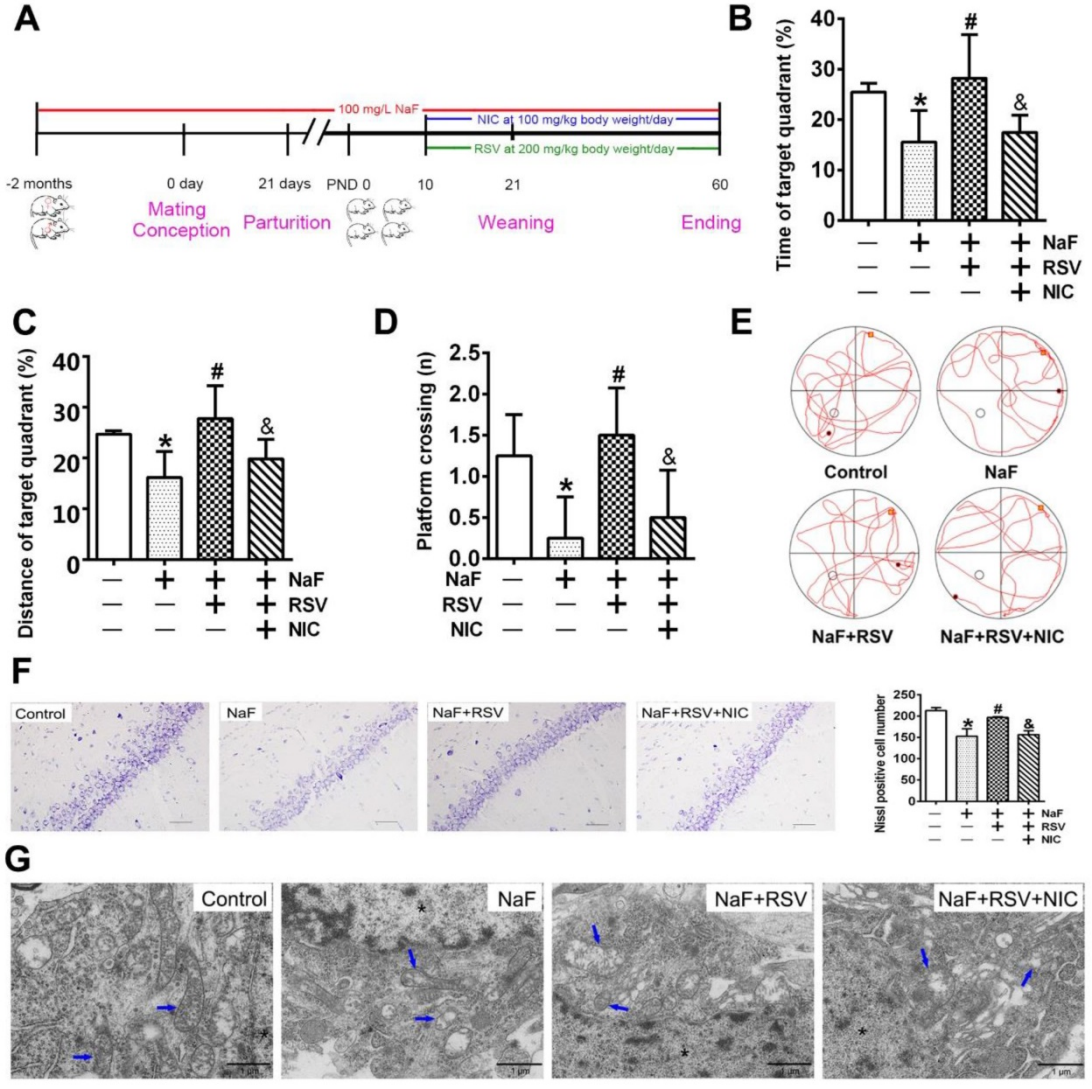
** RSV alleviates neuronal injuries induced by NaF *in vivo.* (A)** Experimental protocol designs of a cohort of offspring rats subjected to NaF, RSV or NIC treatments at various time point. **(B, C)** Time (B) and distance (C) offspring rats spent in the quadrant with the hidden plat form during spatial probe trial of MWM test (*n* = 5 rats per group). **(D, E)** Mean crossing numbers (D) and representative searching traces (E) of offspring rats traveling the target during spatial probe trial of MWM test. **(F)** Representative images of Nissl bodies in hippocampal CA1 region detected using Nissl staining (× 400 magnification) and Nissl-positive cells quantified. Scale bar represents 50 μm, *n* = 2 rats per group. **(G)** Representative images of neuronal mitochondrial ultrastructure of offspring rats shown by TEM (×6000 magnification). Nuclear was indicated as asterisk. Mitochondria were indicated by blue arrows. Scale bars represents 1 μm, *n* = 2 rats per group. Offspring SD rats were developmentally exposed to 100 mg/L NaF from pre-pregnancy until 2 months of delivery, during which 200 mg/kg body weight/day RSV or/and 100 mg/kg body weight/day NIC were administrated from PND10. Data information: Data are presented as mean ± SD. * *P* < 0.05 is considered significant compared with Control, # *P* < 0.05 is significantly different from NaF group and & *P* < 0.05 is considered significant from NaF+RSV group by one-way ANOVA test.

**Figure 7:**
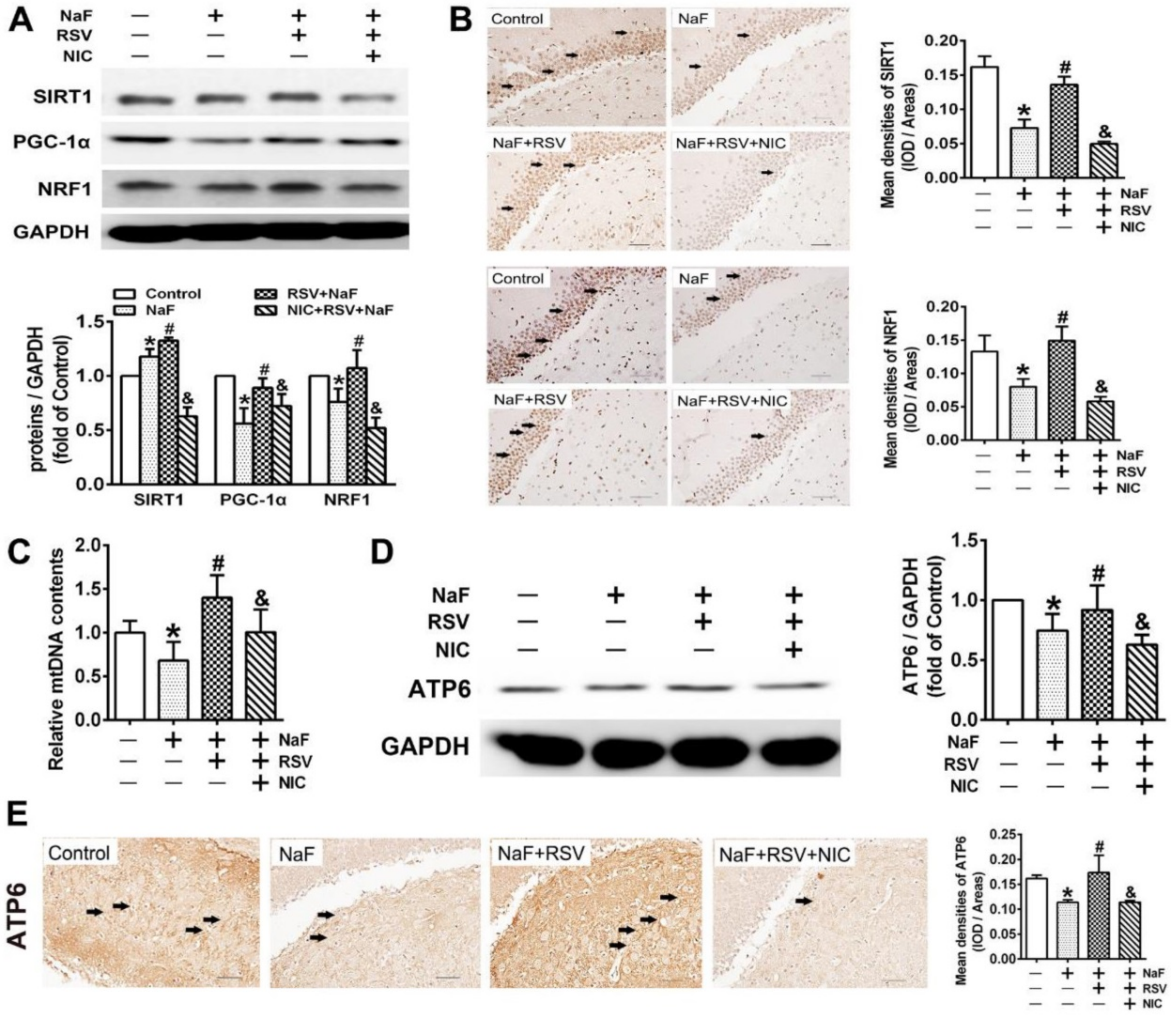
** RSV improves SIRT1-relied mitochondrial biogenesis process in NaF-injured hippocampal tissues of offspring rats. (A)** Immunoblot analyses of SIRT1, PGC-1 and NRF1 in hippocampal tissues (*n* = 3 rats per group). GAPDH was used as the internal control. **(B)** Representative images of the IHC staining for SIRT1^+^ and NRF1^+^ neurons in hippocampal DG region. SIRT1^+^ and NRF1^+^ neuronal cells are demonstrated by black arrows and quantified. Scale bars represent 50 μm, *n* = 2 rats per group. **(C)** RT-qPCR analyses of relative mtDNA contents in hippocampal tissues (*n* = 6 rats per group). **(D)** Immunoblot analyses of ATP6 in hippocampal tissues (*n* = 3 rats per group). **(E)** Representative images of the IHC staining for ATP6^+^ neurons in hippocampal DG region. ATP6^+^ neuronal cells are demonstrated by black arrows and quantified. Scale bars represent 50 μm, *n* = 2 rats per group. Data information: Data are presented as mean ± SD. * *P* < 0.05 is considered significant compared with Control, # *P* < 0.05 is significantly different from NaF group and ^&^*P* < 0.05 is considered significant from NaF+RSV group by one-way ANOVA test.

To further gain convincing evidence in support of RSV's protection against neurotoxic effects of fluoride, we constructed a NaF-exposed rat model with RSV or/and NIC administration. Remarkably, RSV administration significantly relieved the learning and memory impairments of rats exposed to NaF, as displayed by more time and distances rats spent in target quadrant (Figure 6B-C) and increased frequencies of platform crossing (Figure 6D-E) examined by spatial exploration experiment. Meanwhile, RSV reversed the decreased Nissl bodies in hippocampi caused by NaF (Figure 6F). Consistently, abnormal mitochondrial ultrastructure such as swelling and cristae disappearing in hippocampal neurons caused by NaF were effectively attenuated by RSV (Figure 6G). These protective effects of RSV against NaF-induced cognitive impairments and neuronal damage were impeded by NIC supplement. Further, we found that increased SIRT1 protein expression induced by NaF were further elevated by RSV along with augmented protein expression of PGC-1α and NRF1 (Figure 7A), which were also verified by IHC staining (Figure 7B). As expected, impaired mitochondrial biogenesis caused by NaF were evidently alleviated by RSV, as manifested by enhanced mtDNA contents (Figure 7C) and increased protein as well as phenotypic expression of ATP6 (Figure 7D-E). However, with NIC co-treatment, RSV's protection for mitochondrial biogenesis process was suppressed. Collectively, these results demonstrate that RSV activates SIRT1-dependent mitochondrial biogenesis process and promotes subsequent neuronal restoration, thus protecting offspring rats from NaF-induced cognitive impairments.

### Disturbed circulating levels of mitochondrial biogenesis signaling factors are closely correlated with intellectual loss in children with long-term stable fluoride exposure in drinking water

**Figure 8:**
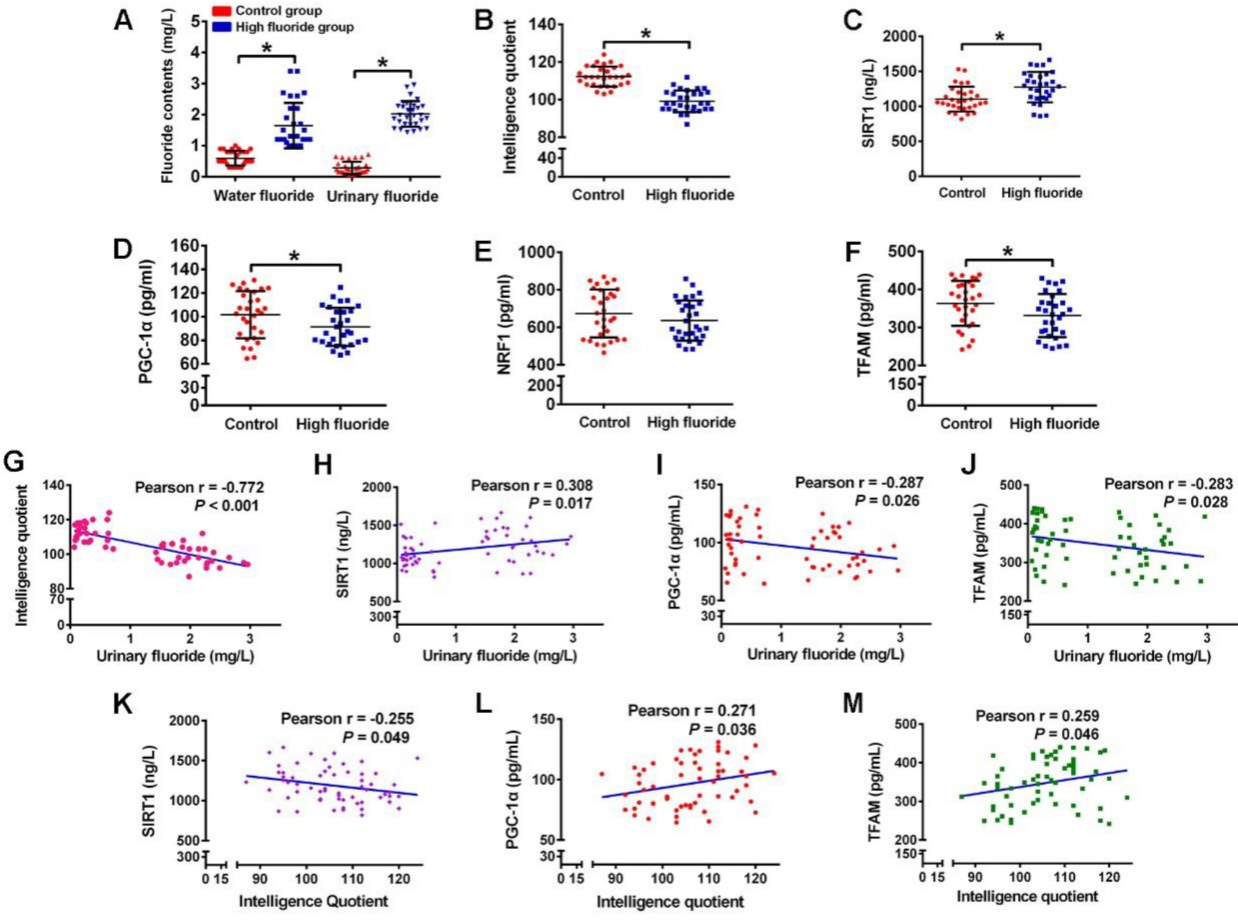
** Disturbance of circulating mitochondrial biogenesis signaling molecules are associated with intelligence loss in children. (A)** Fluoride concentration in drinking water and urine determined by a standardized ion selective electrode method. **(B)** Intelligence quotient (IQ) scores of children measured by CRT-RC2. **(C-F)** Levels of circulating SIRT1 (C), PGC-1α (D), NRF1 (E) and TFAM (F) in peripheral blood lymphocytes detected by ELISA assay. **(G-J)** Correlation between urinary fluoride concentration and IQ scores (G), as well as circulating SIRT1 (H), PGC-1α (I), TFAM (J) levels. **(K-M)** Correlation between IQ scores and circulating SIRT1 (K), PGC-1α (L), TFAM (M) levels. A total of 30 children in control areas and 30 children in high fluoride areas in Tianjin, China were recruited randomly. Data information: Data are presented as mean ± SD. Detailed statistical tests were shown as unpaired two-tailed Student's *t* test (A-F) and Pearson correlation coefficient analysis (G-M). * *P* < 0.05 is considered significant compared with Control.

As measured in children participating in our study, fluoride concentrations in water and urinary fluoride levels were significantly higher, while IQ scores were evidently lower in children from high fluoride group than those from control group (Figure 8A-B). Then we investigated the physiological relevance of mitochondrial biogenesis signaling factors in these children. The circulating SIRT1 levels of children in high fluoride group were evidently higher than those in control group (Figure 8C). Moreover, children's circulating PGC-1α and TFAM levels in high fluoride group showed lower than those in control group (Figure 8D and F). Nevertheless, there was no distinct difference in circulating NRF1 levels between the two groups (Figure 8E). More importantly, children's IQ scores, as well as circulating PGC-1α and TFAM levels, were negatively correlated with urinary fluoride concentrations (Figure 8G, I and J). Inversely, circulating SIRT1 levels were positively correlated with urinary fluoride concentrations (Figure 8H). In addition, there existed a negative correlation between circulating SIRT1 levels and IQ scores (Figure 8K), whereas a positive correlation between circulating levels of PGC-1α and TFAM and IQ scores (Figure 8L-M). These results provide epidemiological evidence that abnormal circulating levels of mitochondrial biogenesis signaling factors are associated with impaired neurodevelopment due to long-term stable fluoride exposure in drinking water.

## Discussion

This study proposed impaired mitochondrial biogenesis as a novel mechanism underlying developmental neurotoxicity induced by chronic long-term fluoride exposure. Notably, RSV improved mitochondrial biogenesis and subsequent mitochondrial function to protect against developmental fluoride neurotoxicity via activating SIRT1-dependent PGC-1α/NRF1/TFAM signaling pathway.

Many identified relevant studies collectively support that fluoride is a developmental neurotoxicant 38. Here, we confirmed the negative correlation between urinary fluoride concentrations and IQ scores in children exposed to stable fluoride concentrations via drinking water. Also, to practically simulate the real children exposure in drinking-water-type fluorosis areas, a rat model of developmental fluoride exposure from pre-pregnancy until 2 months of delivery was constructed. As displayed in our recent study 9, learning and memory abilities of offspring rats were significantly impaired by developmental fluoride exposure, which is in line with previous studies 7, 39. In particular, our study further demonstrated fluoride caused abnormal mitochondrial ultrastructure and dysfunction. These data are consistent with previous observations 40, 41, suggesting developmental fluoride neurotoxicity is closely associated with mitochondrial abnormalities.

Mitochondrial biogenesis is a process by which new mitochondria are produced from existing mitochondria 42. Remarkably, we found mitochondrial biogenesis was impaired following NaF exposure, as demonstrated by decline in mtDNA contents as well as decreased expression of subunits encoded by mtDNA *in vivo* and *in vitro*. As reported, critical regulatory nuclear gene expression profile associated with mitochondrial biogenesis includes PGC-1α, NRF1 and TFAM 43. In this study, PGC-1α/NRF1/ TFAM signaling pathway was evidently impeded by NaF *in vivo* and *in vitro*. We also discovered intense correlations between circulating levels of above mitochondrial biogenesis signaling molecules and intelligence loss in children under chronic long-term fluoride exposure, which has not been reported in previous population study to our knowledge. It is highlighted that impaired mitochondrial biogenesis potentially contributes to mitochondrial dysfunction and has a significant role in the pathogenesis of neurodegenerative diseases 44. Accordingly, TFAM overexpression was adopted to promote mitochondrial biogenesis in cells treated with NaF. Intriguingly, enhanced TFAM expression in cell transfected with Ad-TFAM were further stronger after NaF supplement. Generally, TFAM upregulation in response to stress is considered a possible compensatory mechanism to restore cellular function and homeostasis. Thus, we speculated that TFAM levels were irritable increased as an undue protective response to double stimulations (fluoride and adenovirus treatments simultaneously). Furthermore, in tandem with the restored mitochondrial biogenesis, mitochondrial dysfunction was also attenuated by TFAM overexpression, which ultimately reversing the decline in cell viability induced by NaF. In agreement with our findings, protective effects of TFAM overexpression on cell survival or function are also reported in SH-SY5Y cell-based model of neuropathological conditions 45, 46. Moreover, as examined in children, we found long-term chronic fluoride exposure was associated with decreased PGC-1α and TFAM levels. Overall, these data indicate impeded mitochondrial biogenesis mediated by PGC-1α/NRF1/TFAM signaling pathway results in mitochondrial dysfunction and resultant neuronal death, thus contributing to fluoride neurotoxicity.

SIRT1 has emerged as a crucial regulator of mitochondrial biogenesis by targeting PGC-1α during neurodevelopment 47, 48, which is confirmed by our ChIP-PCR results. Intriguingly, we further observed suppressed SIRT1 deacetylase activity induced by NaF, with opposite enhanced SIRT1 expression at transcriptional and translational levels. The phenomenon fluoride activates SIRT1 also occurs in osteoblast-like MC3T3-E1 cells 49, which is considered as an insufficient protective response to fluoride treatment 23. Nevertheless, contrary to the present work, another study showed the concurrent stimulating effects of fluoride on SIRT1 deacetylase activity and expression in LS8 cells 50. Difference in types of cell lines and fluoride-dosing regimen seem to be responsible for the discrepancies between the two studies. Furthermore, we found long-term chronic fluoride exposure was associated with increased circulating SIRT1 level. Importantly, intelligence loss in children was inversely associated with circulating level of SIRT1. Taken together, these results suggest the expression and activity of SIRT1 are disrupted following fluoride exposure.

RSV, a natural specific SIRT1 activator, is capable of stimulating mitochondrial biogenesis, which has shown great potential as a treatment for a wide range of diseases involving mitochondrial dysfunction 51. In this study, we observed RSV administration restored mitochondrial dysfunction in SH-SY5Y cells, thus protecting them from cell death induced by NaF. Furthermore, impaired mitochondrial biogenesis in NaF-damaged cells were attenuated by RSV. Similar results were obtained in a recent study that demonstrated RSV regulated mitochondrial biogenesis to prevent rotenone-induced neurotoxicity 52. Surprisingly, although we found PGC-1α/ NRF1/TFAM signaling pathway impeded by NaF was strikingly ameliorated by RSV in cells, we did not observe similar changes in SIRT1 protein expression, despite the reinforced SIRT1 deacetylase activity by RSV in NaF-treated cells. Given the widely accepted notion that SIRT1 plays a critical role in protective effects of RSV 53, we used SIRT1 inhibitor NIC to block SIRT1 expression in RSV protection group. Interestingly, NIC supplement suppressed PGC-1α/ NRF1/TFAM signaling pathway and impaired mitochondrial biogenesis, thus reversing the protective effect of RSV against NaF-induced mitochondrial dysfunction and cellular death. Our results are concordant with historical studies where NIC partially attenuates the neuroprotective effects of RSV 54, 55. Supportively, our *in vivo* study further validated that RSV administration activated SIRT1 expression to improve mitochondrial biogenesis and morphology, thus retarding NaF-induced cognitive defects of offspring rats. And all neuroprotective effects of RSV were destroyed by SIRT1 antagonist NIC. Our data support the protective role of RSV in preventing cognitive deficits in neurodegenerative disorders 56. Taken together, these results suggest that RSV protects against developmental fluoride neurotoxicity through activating mitochondrial biogenesis regulated by SIRT1-dependent PGC-1α/ NRF1/TFAM signaling pathway.

**Figure 9:**
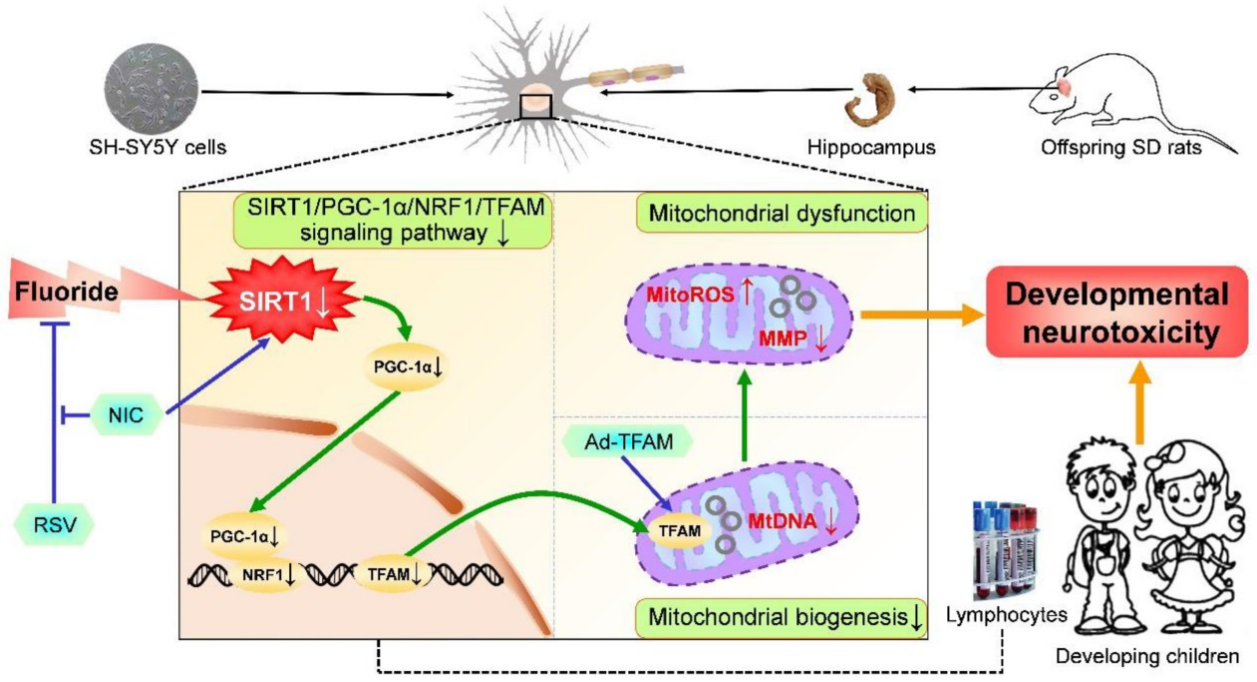
** A proposed model for the role of mitochondrial biogenesis process in developmental fluoride neurotoxicity and protective action of RSV.** Mitochondrial biogenesis process plays a vital role in developmental fluoride neurotoxicity. Improvement in mitochondrial biogenesis by TFAM overexpression causes restoration of mitochondrial function, thus alleviating neurotoxic effects of fluoride. Importantly, RSV protects against developmental fluoride neurotoxicity by enhancing mitochondrial biogenesis and function activated by SIRT1-dependent PGC-1α/NRF1/TFAM signaling pathway, which is suppressed by SIRT1 antagonist NIC. Images of hippocampus, vacuum blood-collection tubes and developmental children were modified from http://p.ayxbk.com/image s/5/5b/Hippocampus_and_seahorse_cropped.JPG; https://www.hellorf.com/image/show/146119724?source=zcool&term=tubes%20prepared, and http://www.jianbihua.cc/renwu/31334_2.html, respectively, with permission.

In summary, we provide *in vivo* and *in vitro* evidence demonstrating that impaired mitochondrial biogenesis regulated by PGC-1α/NRF1/TFAM signaling pathway contributes to developmental neurotoxicity induced by chronic and long-term fluoride exposure. Particularly, we have revealed that RSV possesses protective abilities of promoting mitochondrial biogenesis and mitochondrial function via activating SIRT1-dependent PGC-1α/NRF1/TFAM signaling pathway, thus counteracting developmental neurotoxic effects caused by fluoride (Figure 9). These findings offer new insights into better understanding the mechanisms of developmental fluoride neurotoxicity and a theoretical support for further study of targeting mitochondrial biogenesis via SIRT1 by RSV as a potential dietary therapeutic agent for alleviating fluoride neurotoxicity. In this context, it is worth noting the circulating levels of identified mitochondrial biogenesis signaling molecules in peripheral blood lymphocytes may serve as a predictor for the loss of IQ in children with long-term fluoride exposure, which might represent a new strategy to monitor the developmental neurotoxicity caused by chronic fluoride exposure.

## Supplementary Material

Supplementary figure and tables.

## Abbreviations

Ad-TFAM: TFAM overexpression
ChIP-PCR: Chromatin immunoprecipitation-polymerase chain reaction
CRT-RC2: Combined Raven's Test-The Rural in China
mitoROS: Mitochondrial superoxide
MMP: Mitochondrial membrane potential
mtDNA: mitochondrial DNA
NaF: sodium fluoride
NIC: nicotinamide
NRF1: nuclear respiratory factor 1
PGC-1α: Peroxisome proliferator-activated receptor γ coactivator-1α
PND: postnatal day
RSV: resveratrol
SIRT1: silent information regulator 1
TEM: Transmission electron microscopy
TFAM: mitochondrial transcription factor A
