# Supplementary material for: SIRT1-dependent mitochondrial biogenesis supports therapeutic effects of resveratrol against neurodevelopment damage by fluoride
Source: Theranostics. 2020 Mar 26;10(11):4822–38. doi: 10.7150/thno.42387 (PMC7163447; doi:10.7150/thno.42387)
Supplement: Supplementary file 1 — Supplementary figure and tables. [file thnov10p4822s1.pdf]

Supplementary materials

Tables

Table S1. Sequences of primers used in RT-qPCR for mRNA detections

Gene name	Accession number	Sequence(5'—3')	
PGC-1α	NC_000004.12	F	ACACAGTCGCAGTCACAACAC
		R	GCAGTTCCAGAGAGTTCCACA
NRF1	NC_000007.14	F	CCAGTTTAGTGGGTGGTAGG
		R	CGGGAGCTTTCAAGACATTC
TFAM	NC_000010.11	F	AATGGATAGGCACAGGAAACC
		R	CAAGTATTATGCTGGCAGAAGTC
CO1	NC_012920.1	F	ACGTTGTAGCCCACTTCCAC
		R	TGGCGTAGGTTTGGTCTAGG
CO2	NC_012920.1	F	TACACCGACTACGGCGGACT
		R	AACGTCAAGGAGTCGCAGGT
CO3	NC_012920.1	F	CCCACCAATCACATGCCTAT
		R	GAGGAGCGTTATGGAGTGGA
ATP6	NC_012920.1	F	ACTGCAGGCCACCTACTCAT
		R	TTAAGGCGACAGCGATTTCT
ATP8	NC_012920.1	F	ATACTACCGTATGGCCCACCA
		R	GGGCTTTGGTGAGGGAGGTA
GAPDH	NC_000012.12	F	AGAAGGCTGGGGCTCATTTG
		R	AGGGGCCATCCACAGTCTTC

Table S2. Sequences of primers used in RT-qPCR for measurement of relative mtDNA contents

Gene name	Accession number	Sequence(5'－3')	
ND4	NC_001665.2	F	CTCATCAGTAAGCCATATAGC
		R	TTCGTTCGTAGTTGGTGTT
GAPDH	NC_005103.4	F	GGAAGGACTCATGACCACAGT
		R	GCCATCACGCCACAGTTTC

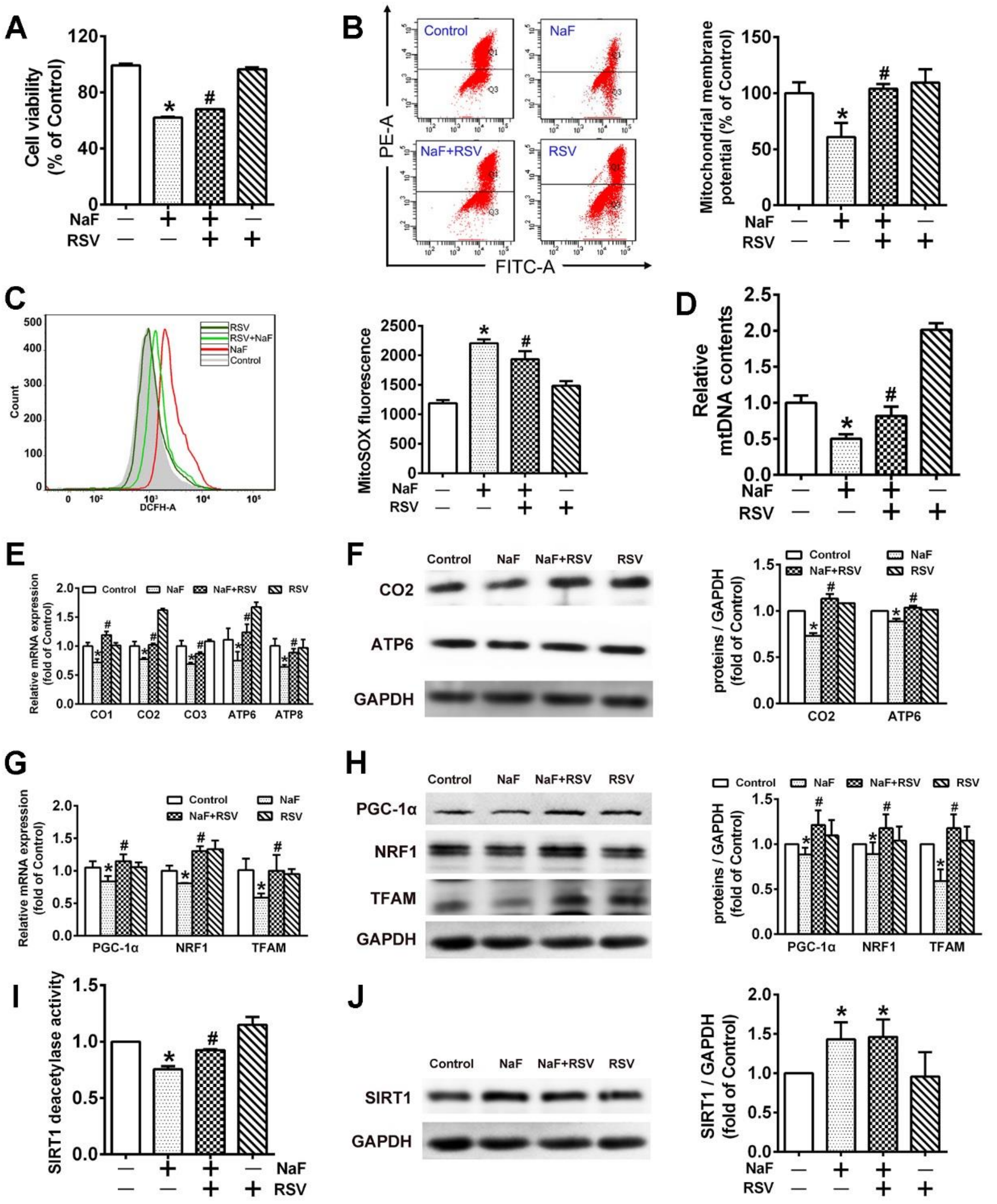

Figure S1. RSV protects against neurotoxic effects of NaF in SHSY5Y cells.

(A) Cell viability determined by CCK-8 assay. **(B, C)** Represent flow plots of MMP levels (B) and mitoROS production (C) by flow cytometry. **(D)** RT-qPCR analysis of relative mtDNA contents. **(E, F)** RT-qPCR (E) and immunoblot (F) analyses of mRNA and protein levels of representative genes encoded by mtDNA. GAPDH was used as the internal control. **(G, H)** RT-qPCR (G) and immunoblot (H) analyses of

mRNA and protein levels of PGC-1α, NFF1 and TFAM. **(I)** SIRT1 deacetylase activity measured by SIRT1 assay kit. **(J)** Immunoblot analysis of SIRT1 expression. SH-SY5Y cells were pretreated with 20 μM RSV and 2 h later were treated with 60 mg/L NaF for 24 h. Data information: Data are presented as mean ± SD. Data were cumulative of at least three independent experiments. * $P < 0.05$ is considered significant compared with Control and # $P < 0.05$ is significantly different from NaF group by one-way ANOVA test.
